# Mass spectrometry-based quantitative proteomic analysis of *Salmonella enterica *serovar Enteritidis protein expression upon exposure to hydrogen peroxide

**DOI:** 10.1186/1471-2180-10-166

**Published:** 2010-06-08

**Authors:** Kihoon Kim, Edward Yang, Gia-Phong Vu, Hao Gong, Jing Su, Fenyong Liu, Sangwei Lu

**Affiliations:** 1 Program in Comparative Biochemistry, University of California, Berkeley, CA 94720, USA; 2 Division of Infectious Diseases and Vaccinology, School of Public Health, University of California, Berkeley, CA 94720, USA

## Abstract

**Background:**

*Salmonella enterica*, a common food-borne bacterial pathogen, is believed to change its protein expression profile in the presence of different environmental stress such as that caused by the exposure to hydrogen peroxide (H_2_O_2_), which can be generated by phagocytes during infection and represents an important antibacterial mechanism of host cells. Among *Salmonella *proteins, the effectors of *Salmonella *pathogenicity island 1 and 2 (SPI-1 and SPI-2) are of particular interest since they are expressed during host infection *in vivo *and are important for invasion of epithelial cells and for replication in organs during systemic infection, respectively. However, the expression profiles of these proteins upon exposure to H_2_O_2 _or to host cells *in vivo *during the established phase of systemic infection have not been extensively studied.

**Results:**

Using stable isotope labeling coupled with mass spectrometry, we performed quantitative proteomic analysis of *Salmonella enterica *serovar Enteritidis and identified 76 proteins whose expression is modulated upon exposure to H_2_O_2_. SPI-1 effector SipC was expressed about 3-fold higher and SopB was expressed approximately 2-fold lower in the presence of H_2_O_2_, while no significant change in the expression of another SPI-1 protein SipA was observed. The relative abundance of SipA, SipC, and SopB was confirmed by Western analyses, validating the accuracy and reproducibility of our approach for quantitative analysis of protein expression. Furthermore, immuno-detection showed substantial expression of SipA and SipC but not SopB in the late phase of infection in macrophages and in the spleen of infected mice.

**Conclusions:**

We have identified *Salmonella *proteins whose expression is modulated in the presence of H_2_O_2_. Our results also provide the first direct evidence that SipC is highly expressed in the spleen at late stage of salmonellosis *in vivo*. These results suggest a possible role of SipC and other regulated proteins in supporting survival and replication of *Salmonella *under oxidative stress and during its systemic infection *in vivo*.

## Background

*Salmonella enterica *is one of the leading causes of food-borne illnesses around the world [1,2]. There are two major serotypes of *Salmonella enterica*, namely *Salmonella enterica *serovar Enteritidis (*S. Enteritidis*) and Typhimurium (*S. Typhimurium*). In recent years, *S. Enteritidis *represents one of the most commonly reported serotypes associated with food poisoning illness in the United States [3]. Two hallmarks of *Salmonella *pathogenesis are the invasion of non-phagocytic cells such as the epithelial cells of the intestinal mucosa, and the survival inside macrophages during systemic infection. The mechanisms of both processes are linked to the functions of two type III secretion systems (T3SS) of *Salmonella *that are encoded and regulated by a cluster of genes at the *Salmonella *Pathogenicity Island 1 and 2 (SPI-1 and SPI-2), respectively. It is believed that SPI-1 T3SS is responsible for invasion of non-phagocytic cells, while SPI-2 T3SS is essential for intracellular replication and systemic infection [4,5].

In order to survive and replicate in an aerobic environment, organisms including *Salmonella *must cope with reactive oxygen species such as hydrogen peroxide (H_2_O_2_), which are formed in respiring cells as incomplete reduction products of molecular oxygen, and which can cause damage to DNA, RNA, protein, and lipids [6-8]. To respond to oxidative stress, bacteria activate a set of globally regulated genes, including two known stimulons: peroxide stimulons and superoxide stimulons [7,9-12].

The response of *Salmonella *to oxidative stress represents a key component of its pathogenesis [7,9]. Reactive oxygen species generated by the NADPH phagocytic oxidase system in phagocytes play an important role in controlling *Salmonella *replication in macrophages and systemic infection in the spleen [13,14]. To combat the damaging effects of this oxidative stress and survive in macrophages during systemic infection such as in the spleen, it is believed that *Salmonella *uses unique strategies and expresses specific proteins to carry out defense and repair functions [7,9]. While little is known about the expression of SPI-1 factors upon oxidative stress, several SPI-1 factors SipA, SopA, SopB, SopD, and SopE2 of *S. Typhimurium *were found to be expressed in the spleen of infected animals at the late stages of infection when *Salmonella *is believed to replicate in splenic macrophages [15,16]. These results suggest that in addition to their generally recognized roles in invasion, the SPI-1 factors may also play an important role post-invasion, including a possible role in resistance to the oxidative stress generated by tissue macrophages. An understanding of the expression profiles of *Salmonella *SPI-1 factors and other proteins in the presence of reactive oxygen species such as H_2_O_2 _should provide insight into the identification of virulent determinants important for *Salmonella *to survive in macrophages and cause systemic infection in the spleen *in vivo*.

The expression of *Salmonella *genes (including those encoding SPI-1 factors) *in vitro *under various conditions has been extensively studied [17-21]. However, most of these studies were performed by examining the transcription levels of *Salmonella *genes either using microarray or a reporter system [17,19-23]. Recently, proteomic analysis of *Salmonella *protein expression in the spleen of infected animals has been reported [24]. Furthermore, Smith and co-workers have reported global protein profiles of *Salmonella enterica *serovars Typhimurium and Typhi cultured at the stationary phase, logarithmic (log) phase, or phagosome-mimicking culture conditions, and the expression profiles of proteins in infected macrophages [25-28]. However, to our knowledge, global expression profiling of *Salmonella *proteins upon exposure to reactive oxygen species such as H_2_O_2 _has not been reported, and efforts to identify proteins whose expression levels are affected by oxidative stress have been limited mostly to a few proteins at a time [9,29,30]. In addition, expression of *Salmonella *proteins including those of SPI-1 *in vivo *during the established phase of infection has not been extensively studied.

In this study, we have modified the procedure of Stable Isotope Labeling by Amino acids in Cell culture (SILAC) [31,32] to develop a mass spectrometry (MS)-based approach to carry out quantitative proteomic analysis of *Salmonella*. Using this procedure, we have identified 76 proteins from a strain of *Salmonella enterica *serovar Enteritidis that are differentially regulated upon exposure to H_2_O_2_. The results on selected SPI-1 proteins were confirmed by Western blot analyses, validating the accuracy and reproducibility of our approach for quantitative analyses of protein expression. The expression of several SPI-1 proteins was further analyzed in infected macrophages and in the spleen of infected mice. These results suggest a possible role for SPI-1 proteins in *Salmonella *infection in the presence of oxidative stress and in systemic infection in an animal host.

## Results

### Stable isotope labeling of *Salmonella *with ^15^N-containing growth media

**Figure 1:**
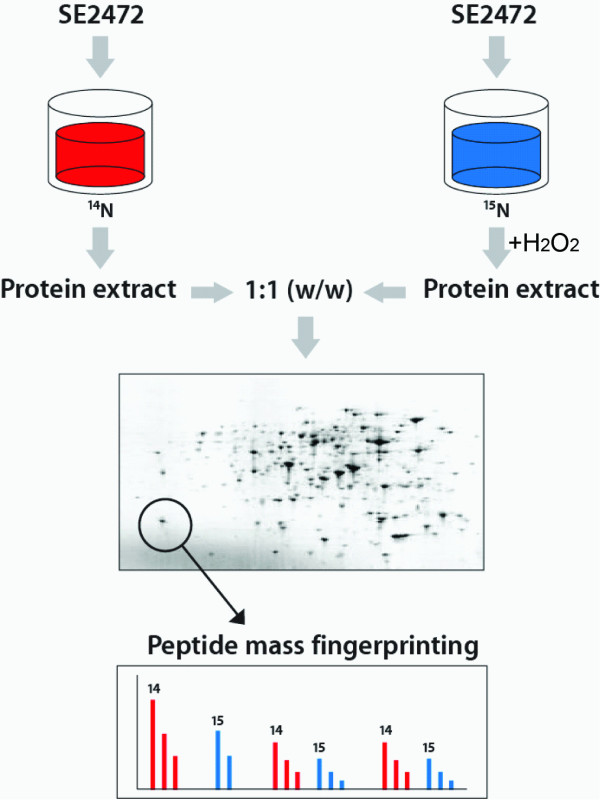
**Schematic representation of metabolic labeling of *Salmonella *with the ^15^N isotope**.

We used a virulent clinical isolate of *Salmonella enterica *serovar Enteritidis SE2472 for this analysis. Our previous studies have shown that almost all clinical strains analyzed, including SE2472, exhibited similar levels of resistance to H_2_O_2 _[33]. We chose this strain to examine the expression profiles of *Salmonella *proteins upon exposure to H_2_O_2_, a stress condition relevant to what *Salmonella *may encounter in macrophages and in the spleen during bacterial infection *in vivo*. To determine protein levels in two or more different biological states (e.g. in the absence and presence of H_2_O_2_), we modified the SILAC procedure (Figure 1) in which the introduction of a stable isotope ^15^N into the protein mixture provides a means to quantitatively analyze two sets of protein mixtures simultaneously [31,32]. Stable isotope-based quantification relies on the premise that the relative signal intensity of two analytes that are chemically identical but different in stable isotope compositions can be resolved in a mass spectrometer, thus giving a true measure of the relative abundance of the analytes [31,32,34,35]. To determine the efficiency of the labeling and incorporation of the heavy isotope, SE2472 was grown in ^15^N-containing LB broth-like media. SE2472 appeared to grow in the normal (^14^N) and ^15^N-containing LB broth-like media as well as in the LB broth as they reach similar titers in these media (data not shown). Bacteria were harvested at different time points and the extent of ^15^N-labeling of *Salmonella *proteins was examined by MS analysis in comparison to the control ^14^N labeled bacteria. Growth in ^15^N-labeled media for 6 hours or more was sufficient to label the entire *Salmonella *proteome with ^15^N (data not shown). The proteins examined and all the peptides of each protein appeared to have identical incorporation rate. Accordingly, all labeling experiments were carried out for at least 6 hours in this study.

### Wild type-like growth phenotypes of labeled bacteria

**Figure 2:**
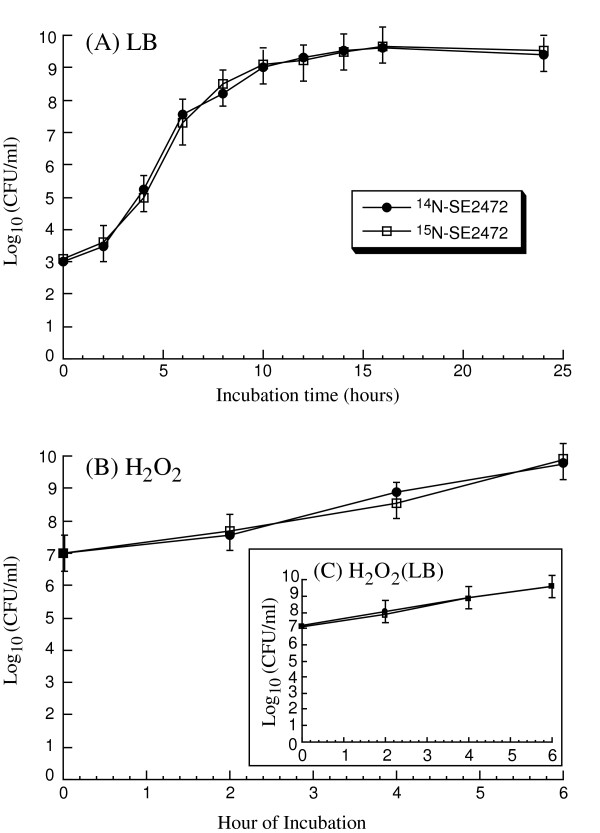
**Growth analysis of *S. Enteritidis *SE2472**. (A) Growth of normal (^14^N) and ^15^N-labeled *S. Enteritidis *SE2472 in LB broth. (B) The survival of normal (^14^N) and ^15^N-labeled *Salmonella *grown in LB broth-like labeling media after exposure to H_2_O_2_, compared to the survival of the same cultures grown in LB broth after exposure to H_2_O_2 _(inset).

One of our main objectives in the study was to use the expression of the labeled proteins to monitor *Salmonella *protein levels when *Salmonella *is exposed to oxidative stress. Thus, it is necessary to determine whether ^15^N-labeled *Salmonella *retain the growth and oxidative stress-resistant properties of the unlabeled SE2472 *in vitro*. ^15^N-labeled *Salmonella *appeared to grow as well as the unlabeled bacteria in LB broth (Figure 2A). No detectable difference in the colony size and morphology was observed between these two cultures. Furthermore, no difference was detected between the survival of the N^14^- and N^15^-labeled bacteria in either the LB broth-like labeling media or the LB broth in the presence of 5 mM H_2_O_2_, a concentration well below the minimal inhibition concentration (MIC) of SE2472 (20 mM) but substantially above the natural extracellular environment (Figure 2B).

### Quantitative proteomic analyses of *Salmonella *protein expression

**Figure 3:**
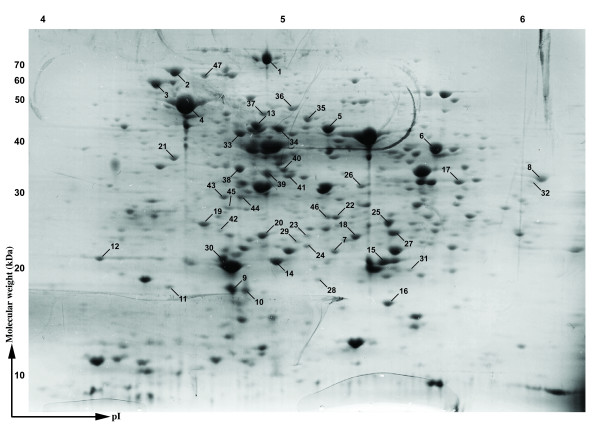
**Two-dimensional gel electrophoresis of *S. Enteritidis *SE2472 total proteins**. Approximately 200 μg of total SE2472 proteins were loaded onto a 2 D gel and visualized by the silver staining method.

To investigate the expression profiles of *Salmonella *upon exposure to oxidative stress, single colonies of SE2472 were grown in ^14^N- or ^15^N-containing LB broth-like media and only the ^15^N culture was treated with 5 mM H_2_O_2 _for 2 hours to simulate the oxidative stress condition (Figure 1). Total proteins from the ^14^N- and ^15^N- samples were extracted and quantified. A 1:1 (by weight) mixture of two samples was prepared and 200 μg of total proteins were separated by two-dimensional (2-D) gel electrophoresis. Visualization by silver staining revealed approximately 200 protein spots across the pI and molecular weight range of the gel, which were further investigated using quantitative proteomics (Figure 3).

**Figure 4:**
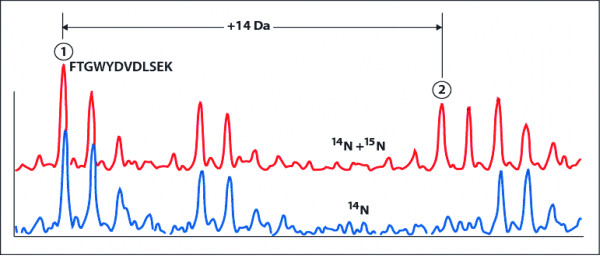
**Quantitation of proteins by their tryptic peptide fragments**. A tryptic peptide fragment FTGWYDVDLSEK (MW 1459.81) from *S. Enteritidis *phosphoglyceromutase was analyzed for the level of downregulation. A peak at m/z 1473 represents ^15^N-labeled population (upper spectrum), which does not appear in the unlabeled population. The ratio of two peak intensities (27 and 17, respectively) represents a relative protein expression level of 0.6, or a 40% downregulation.

Analysis with matrix-assisted laser desorption/ionisation-time of flight (MALDI-ToF) mass spectrometry was performed to map tryptic fragments from the mixture of the ^14^N- and ^15^N-(unexposed and H_2_O_2_-exposed) samples, where two sets of peptide fingerprints appear on the same spectrum (Figure 4, Table 1). We distinguished the two sets of peaks by initially using the ^14^N peaks to identify the protein and amino acid contents of each peak (Figure 4 and Table 1), then using peak information to deduce the location of the ^15^N peaks. The ratio of the peak heights (^15^N/^14^N) was then used for relative quantification (Figures 1 and 4). Figure 4 shows an example taken from a protein sample, a tryptic peptide fragment FTGWYDVDLSEK (MW 1459.81) from *S. Enteritidis *phosphoglyceromutase. A peak at m/z 1473 represents the ^15^N-labeled population (Figure 4, upper spectrum), which does not appear in the unlabeled population. The ratio of two peak intensities (27 and 17, respectively) represents a relative protein expression level of 0.6, or a 40% downregulation. To further increase the accuracy of our results, each set of experiments was repeated three times. Only those proteins that were detected and identified with high confidence in all three independent experiments are listed in Table 2.

**Table 1:** MALDI-ToF analysis and identification of SE2472 proteins.

Locus Tag	Description	Gene	Mass (KDa)	pI	Coverage
PSLT011	Dlp (SrgA)	srgA	24.74	8.58	38%
STM0007	Transaldolase B	talB	35.15	5.09	19%
STM0012	Chaperone protein *dnaK *(Heat shock protein 70)	dnaK	69.2	4.84	22%
STM0013	Chaperone protein *dnaJ*	dnaJ	41.31	8.41	25%
STM0093	Organic solvent tolerance protein	Imp	89.8	5.21	23%
STM0102	L-arabinose isomerase	araA	55.89	5.88	23%
STM0158	Aconitate hydratase 2	acnB	82.2	5.35	29%
STM0217	Elongation factor Ts	tsf	33.18	5.16	41%
STM0316	Aminoacyl-histidine dipeptidase	pepD	52.69	5.17	15%
STM0432	Phosphonoacetaldehyde hydrolase	phnX	28.57	5.58	41%
STM0435	Nucleotide-binding protein	yajQ	18.31	5.6	52%
STM0447	Trigger factor	tig	48.02	4.84	23%
STM0488	Adenylate kinase	adk	23.49	5.53	51%
STM0536	Peptidyl-prolyl cis-trans isomerase B	ppiB	18.13	5.52	45%
STM0608	Chain T, crystal structure of Ahpc	ahpC	20.64	5.03	24%
STM0730	Citrate synthase	gltA	48.11	6.35	24%
STM0772	Phosphoglyceromutase	gpmA	28.48	5.78	19%
STM0776	UDP-galactose 4-epimerase	galE	37.28	5.79	31%
STM0781	Molybdate transporter periplasmic protein	modA	27.5	6.53	67%
STM0794	Biotin synthase	bioB	38.8	5.42	53%
STM0830	Glutamine-binding periplasmic protein precursor	glnH	27.23	8.74	67%
STM0877	Putrescine-binding periplasmic protein precursor	potF	41	6.02	35%
STM0999	Outer membrane protein F precursor	ompF	40.05	4.73	28%
STM1091	Secretory Effector Protein	SopB	61.93	9.27	42%
STM1220	N-acetyl-D-glucosamine kinase	nagK	33.06	5.09	29%
STM1231	DNA-binding response regulator in PhoQ system	phoP	25.61	5.28	33%
STM1290	Glyceraldehyde-3-phosphate dehydrogenase	gapA	36.1	6.33	29%
STM1296	Putative oxidoreductase	ydjA	20.13	6.75	29%
STM1302	Exonuclease III	xthA	30.79	6.19	23%
STM1303	Succinylornithine transaminase	astC	43.72	6.13	34%
STM1310	NAD synthetase	nadE	30.57	5.36	27%
STM1378	Pyruvate kinase I	pykF	50.66	5.66	31%
STM1431	Superoxide dismutase	sodB	21.35	5.58	35%
STM1544	PhoPQ-regulated protein	pqaA	59.27	6.87	20%
STM1567	Alcohol dehydrogenase	adhP	35.49	5.8	42%
STM1589	Putative NADP-dependent oxidoreductase	yncB	39.2	5.6	23%
STM1641	ATP-dependent helicase	hrpA	148.71	8.22	15%
STM1661	Putative universal stress protein	ydaA	35.62	5.17	66%
STM1682	Thiol peroxidase	tpx	18.19	4.93	54%
STM1714	DNA topoisomerase I	topA	97.03	8.56	26%
STM1727	Tryptophan synthase	trpA	28.65	5.28	20%
STM1746.S	Chain A, structural basis of multispecificity in Oppa	oppA	58.77	5.85	29%
STM1796	Trehalase, periplasmic	treA	63.6	5.19	63%
STM1886	Glucose-6-phosphate 1-dehydrogenase	zwf	55.92	5.52	26%
STM1923	Chemotaxis protein *motA*	motA	32.08	5.47	31%
STM1954	Cystine-binding periplasmic protein precursor	fliY	28.79	8.81	23%
STM1959	Flagellin	fliC	51.62	4.79	56%
STM2104	Phosphomannomutase in colanic acid gene cluster	cpsG	50.02	5.18	20%
STM2167	NADH independent D-lactate dehydrogenase	dld	65.05	6.47	31%
STM2190	D-galactose binding periplasmic protein	mglB	35.81	5.81	31%
STM2203	Endonuclease IV	nfo	31.2	5.17	45%
STM2205	Fructose-1-phosphate kinase	fruK	33.71	5.36	39%
STM2282	Glycerophosphodiester phosphodiesterase	glpQ	40.42	5.66	24%
STM2337	Acetate kinase	ackA	43.26	5.93	21%
STM2347	Putative phosphoesterase	yfcE	19.91	5.93	43%
STM2362	Amidophosphoribosyltransferase	purF	56.56	5.51	23%
STM2501	Polyphosphate kinase	ppk	80.46	8.7	30%
STM2549	Anaerobic sulfide reductase	asrB	30.61	6.24	28%
STM2647	Uracil-DNA glycosylase	ung	25.48	6.56	67%
STM2829	DNA strand exchange and recombinant protein	recA	37.94	5.08	28%
STM2864	Iron transporter protein, *fur *regulated	sitD	33.7	7.84	41%
STM2882	Secretory Effector Protein	sipA	73.94	6.41	35%
STM2884	Translocation Machinery Component	sipC	42.98	8.88	38%
STM2924	RNA polymerase sigma factor *rpoS*	rpoS	37.93	4.86	29%
STM2952	Enolase	eno	36.24	5.13	30%
STM2976	L-fucose isomerase	fucI	64.77	5.6	31%
STM2993	Exonuclease V, alpha chain	recD	67.05	8.02	36%
STM3068	Fructose-bisphosphate aldolase	fba	39.3	5.68	25%
STM3069	Phosphoglycerate kinase	pgk	41.28	5.09	38%
STM3186	Outer membrane channel protein	tolC	53.39	5.42	31%
STM3219	2,4-dieonyl-CoA reductase	fadH	73.13	6.55	35%
STM3225	Serine/threonine transporter	sstT	43.41	8.43	33%
STM3294	Phosphoglucosamine mutase	glmM	47.44	5.74	32%
STM3342	Stringent starvation protein A	sspA	32.05	5.22	19%
STM3359	Malate dehydrogenase	mdh	32.63	6.01	22%
STM3380	Acetyl CoA carboxylase	accC	49.26	6.52	28%
STM3401	Shikimate dehydrogenase	aroE	29.29	5.73	51%
STM3445	Elongation factor Tu	tuf	43.26	5.3	32%
STM3446	Elongation factor G	fusA	77.72	5.17	23%
STM3484	DNA adenine methylase	dam	32.03	8.93	26%
STM3496	Putative hydrolase	yrfG	72.4	5.23	19%
STM3500	Phosphoenolpyruvate carboxykinase	pckA	59.9	5.67	28%
STM3502	Osmolarity response regulator	ompR	27.35	6.04	31%
STM3557	Glycerol-3-phosphatase transporter binding protein	ugpB	48.49	6.97	15%
STM3612	2-dehydro-3-deoxygluconokinase	kdgK	34.35	5.01	17%
STM3884	D-ribose periplasmic binding protein	rbsB	30.9	8.54	38%
STM3968	Uridine phosphorylase	udp	27.38	6.32	34%
STM3997	Thiol:disulfide interchange protein	dsbA	22.9	6.3	54%
STM4029	Putative acetyltransferase	yiiD	36.92	6.08	34%
STM4166	NADH pyrophosphatase	nudC	29.62	5.89	48%
STM4256	Single-strand DNA-binding protein	ssb	19.06	5.46	34%
STM4329	Co-chaperonin *groES*	groES	10.19	5.36	56%
STM4330	Chaperonin *groEL*	groEL	57.16	4.85	38%
STM4343	Fumarate reductase	frdA	65.49	5.95	19%
STM4359	DNA mismatch repair protein *mutL*	mutL	67.76	6.51	21%
STM4414	Inorganic pyrophosphatase	ppa	19.68	5.01	43%
STM4513	Putative permease	yjiG	16.12	7.76	61%
STM4567	Deoxyribose-phosphate aldolase	deoC	27.68	5.87	47%
STM4568	Thymidine phosphorylase	deoA	47	4.96	38%
STM4569	Phosphopentomutase	deoB	44.24	5.15	52%
STM4598	Two-component response regulator	arcA	45.56	5.47	58%
STY2300	CDP-6-deoxy-D-xylo-4-hexulose-3-dehydrase	rfbH	48.1	5.27	46%
STY2300	CDP-4-keto-6-deoxy-D-glucose-3-dehydrase	ddhC	48.2	5.35	39%

**Table 2:** Quantitative analysis of the expression of SE2472 proteins upon exposure to H_2_O_2_.

Locus Tag	Description	Gene	% Change
PSLT011	Dlp (SrgA)	srgA	12 ± 2%
STM0007	Transaldolase B	talB	0%
STM0012	Chaperone protein *dnaK *(Heat shock protein 70)	dnaK	56 ± 7%
STM0013	Chaperone protein *dnaJ*	dnaJ	38 ± 3%
STM0093	Organic solvent tolerance protein	Imp	210 ± 30%
STM0102	L-arabinose isomerase	araA	26 ± 2%
STM0158	Bifunctional aconitate hydratase	acnB	25 ± 5%
STM0217	Elongation factor Ts	tsf	21 ± 4%
STM0316	Aminoacyl-histidine dipeptidase	pepD	9 ± 1%
STM0432	Phosphonoacetaldehyde hydrolase	phnX	31 ± 3%
STM0435	Nucleotide-binding protein	yajQ	0%
STM0447	Trigger factor	tig	11 ± 2%
STM0488	Adenylate kinase	adk	0%
STM0536	Peptidyl-prolyl cis-trans isomerase B	ppiB	0%
STM0608	Chain T, crystal structure of Ahpc	ahpC	0%
STM0730	Citrate synthase	gltA	42 ± 5%
STM0772	Phosphoglyceromutase	gpmA	-40 ± 10%
STM0776	UDP-galactose 4-epimerase	galE	23 ± 2%
STM0781	Molybdate transporter periplasmic protein	modA	11 ± 2%
STM0794	Biotin synthase	bioB	0%
STM0830	Glutamine-binding periplasmic protein precursor	glnH	10 ± 3%
STM0877	Putrescine-binding periplasmic protein precursor	potF	11 ± 2%
STM0999	Outer membrane protein F precursor	ompF	0%
STM1091	Secretory Effector Protein	SopB	-55% ± 7%
STM1220	N-acetyl-D-glucosamine kinase	nagK	12 ± 3%
STM1231	DNA-binding response regulator in PhoQ system	phoP	20 ± 6%
STM1290	Glyceraldehyde-3-phosphate dehydrogenase	gapA	31 ± 3%
STM1296	Putative oxidoreductase	ydjA	-30 ± 5%
STM1302	Exonuclease III	xthA	0%
STM1303	Succinylornithine transaminase	astC	41 ± 7%
STM1310	NAD synthetase	nadE	9 ± 1%
STM1378	Pyruvate kinase I	pykF	87 ± 12%
STM1431	Superoxide dismutase	sodB	110 ± 20%
STM1544	PhoPQ-regulated protein	pqaA	19 ± 2%
STM1567	Alcohol dehydrogenase	adhP	9 ± 2%
STM1589	Putative NADP-dependent oxidoreductase	yncB	12 ± 2%
STM1641	ATP-dependent helicase	hrpA	20 ± 3%
STM1661	Putative universal stress protein	ydaA	140 ± 20%
STM1682	Probable peroxidase	tpx	19 ± 2%
STM1714	DNA topoisomerase I	topA	17 ± 4%
STM1727	Tryptophan synthase	trpA	37 ± 9%
STM1746.S	Chain A, structural basis of multispecificity in Oppa	oppA	0%
STM1796	Trehalase, periplasmic	treA	25 ± 3%
STM1886	Glucose-6-phosphate 1-dehydrogenase	zwf	0%
STM1923	Chemotaxis protein *motA*	motA	14 ± 3%
STM1954	Cystine-binding periplasmic protein precursor	fliY	9 ± 2%
STM1959	Flagellin	fliC	0%
STM2104	Phosphomannomutase in colanic acid gene cluster	cpsG	23 ± 6%
STM2167	NADH independent D-lactate dehydrogenase	dld	16 ± 5%
STM2190	D-galactose binding periplasmic protein	mglB	34 ± 3%
STM2203	Endonuclease IV	nfo	0%
STM2205	Fructose-1-phosphate kinase	fruK	35 ± 3%
STM2282	Glycerophosphodiester phosphodiesterase	glpQ	15 ± 3%
STM2337	Acetate kinase	ackA	23 ± 3%
STM2347	Putative phosphoesterase	yfcE	0%
STM2362	Amidophosphoribosyltransferase	purF	10 ± 4%
STM2501	Polyphosphate kinase	ppk	7 ± 3%
STM2549	Anaerobic sulfide reductase	asrB	0%
STM2647	Uracil-DNA glycosylase	ung	27 ± 2%
STM2829	DNA strand exchange and recombinant protein	recA	24 ± 2%
STM2864	Iron transporter protein, fur regulated	sitD	-45 ± 8%
STM2882	Secretory Effector Protein	sipA	0%
STM2884	Translocation Machinery Component	sipC	301 ± 30%
STM2924	RNA polymerase sigma factor *rpoS*	rpoS	13 ± 2%
STM2952	Enolase	eno	23 ± 4%
STM2976	L-fucose isomerase	fucI	0%
STM2993	Exonuclease V, alpha chain	recD	0%
STM3068	Fructose-bisphosphate aldolase	fba	52 ± 7%
STM3069	Phosphoglycerate kinase	pgk	20 ± 3%
STM3186	Outer membrane channel protein	tolC	0%
STM3219	2,4-dieonyl-CoA reductase	fadH	24 ± 3%
STM3225	Serine/threonine transporter	sstT	23 ± 3%
STM3294	Phosphoglucosamine mutase	glmM	18 ± 2%
STM3342	Stringent starvation protein A	sspA	-20 ± 4%
STM3359	Malate dehydrogenase	mdh	36 ± 6%
STM3380	Acetyl CoA carboxylase	accC	11 ± 2%
STM3401	Shikimate dehydrogenase	aroE	12 ± 3%
STM3445	Elongation factor Tu	tuf	0%
STM3446	Elongation factor G	fusA	9 ± 2%
STM3484	DNA adenine methylase	dam	26 ± 3%
STM3496	Putative hydrolase	yrfG	0%
STM3500	Phosphoenolpyruvate carboxykinase	pckA	330 ± 40%
STM3502	Osmolarity response regulator	ompR	12 ± 3%
STM3557	Glycerol-3-phosphatase transporter binding protein	ugpB	0%
STM3612	2-dehydro-3-deoxygluconokinase	kdgK	9 ± 2%
STM3884	D-ribose periplasmic binding protein	rbsB	31 ± 3%
STM3968	Uridine phosphorylase	udp	11 ± 5%
STM3997	Thiol:disulfide interchange protein	dsbA	10 ± 5%
STM4029	Putative acetyltransferase	yiiD	0%
STM4166	NADH pyrophosphatase	nudC	10 ± 2%
STM4256	Single-strand DNA-binding protein	ssb	19 ± 2%
STM4329	Co-chaperonin *groES*	groES	51 ± 3%
STM4330	Chaperonin *groEL*	groEL	43 ± 2%
STM4343	Fumarate reductase	frdA	40 ± 2%
STM4359	DNA mismatch repair protein mutL	mutL	41 ± 3%
STM4414	Inorganic pyrophosphatase	ppa	0%
STM4513	Putative permease	yjiG	-78 ± 15%
STM4567	Deoxyribose-phosphate aldolase	deoC	0%
STM4568	Thymidine phosphorylase	deoA	-9 ± 2%
STM4569	Phosphopentomutase	deoB	0%
STM4598	Two-component response regulator	arcA	10 ± 4%
STY2300	CDP-6-deoxy-D-xylo-4-hexulose-3-dehydrase	rfbH	0%
STY2300	CDP-4-keto-6-deoxy-D-glucose-3-dehydrase	ddhC	0%

Relative expression level in the presence of H_2_O_2 _compared against control (in the absence of H_2_O_2_) is shown. An average of 10 peaks was used to calculate the mean intensity ratios and the error percentage of each protein spot. The results were the average from triplicate experiments. The limit of detection was arbitrarily set to 5% and any proteins that showed less than 5% change were classified as 0%.

### Automation of expression profiling

Predicting the location of ^15^N peaks for any given peptide was a two-step process. First, the protein was identified and the amino acid contents of each peak were searched using the MASCOT software. Based on the number of nitrogen atoms in the peptide, a prediction was then made as to the molecular weight of the ^15^N peaks. To facilitate this process, we developed a custom VBScript for Microsoft Excel that automatically predicts ^15^N peak locations with a simple copy and paste feature. Using this script, many sets of peak data can be processed within minutes, virtually eliminating the need to manually inspect each peak for the number of nitrogen atoms.

Using the developed program, we generated a list of 103 SE2472 proteins and their expression profiles upon exposure to H_2_O_2 _(Table 1 and 2). All these proteins were detected and identified with high confidence in all three independent experiments. We should note that the absence of a protein in our results does not necessarily mean it was not expressed and/or induced; instead its expression status is yet to be determined. The majority of protein expression was up-regulated, albeit at different levels. We further categorized proteins into different groups based on their functions, as shown in Table 3. Interestingly, SipC and SopB, which are the SPI-1 translocase and effector, were differentially expressed in the presence of H_2_O_2_. SipC was about 3-fold higher and SopB was 2-fold lower in the exposed samples, while no significant change in the expression of another SPI-1 protein SipA was observed (Table 2 and 3).

**Table 3:** Expression proteomics of SE2472 upon exposure to H_2_O_2_, categorized by protein functions.

Description	Change
**Glycolysis/Gluconeogenesis**	
Enolase	23 ± 4%
Fructose-1-phosphate kinase	35 ± 3%
Fructose-bisphosphate aldolase	52 ± 7%
Phosphoenolpyruvate carboxykinase	330 ± 40%
Phosphoglycerate kinase	20 ± 3%
Phosphoglyceromutase	-40 ± 10%
Phosphopyruvate hydratase	12 ± 2%
Pyruvate kinase I	87 ± 12%

**TCA Cycle**	
Aconitate hydratase 2	18 ± 2%
Bifunctional aconitate hydratase	25 ± 5%
Citrate synthase	42 ± 5%
Malate dehydrogenase	36 ± 6%

**Transcription/Translation**	
Elongation factor G	9 ± 2%
Elongation factor Ts	21 ± 4%
Elongation factor Tu	0%
Endonuclease IV	0%
RNA polymerase sigma factor *rpoS*	13 ± 2%

**DNA Replication/Repair**	
ATP-dependent helicase	20 ± 3%
DNA adenine methylase	26 ± 3%
DNA mismatch repair protein mutL	41 ± 3%
Single-strand DNA-binding protein	19 ± 2%
Uracil-DNA glycosylase	27 ± 2%

**Type III Secretion System**	
Secretory Effector Protein (SipA)	0%
Translocation Machinery Component (SipC)	301 ± 30%
Secretory Effector Protein (SopB)	-55% ± 7%

**Pentose Phosphate Pathway**	
Deoxyribose-phosphate aldolase	0%
Glucose-6-phosphate 1-dehydrogenase	0%
Phosphopentomutase	0%
2-dehydro-3-deoxygluconokinase	9 ± 2%

**Nucleotide synthesis and metabolism**	
Amidophosphoribosyltransferase	10 ± 4%
Thymidine phosphorylase	-9 ± 2%
Uridine phosphorylase	11 ± 5%

**Amino acid synthesis and metabolism**	
Shikimate dehydrogenase	12 ± 3%
Succinylornithine transaminase	41 ± 7%
Tryptophan synthase	37 ± 9%

Representative proteins are shown.

### Validation of differential expression of the SPI-1 proteins

**Figure 5:**
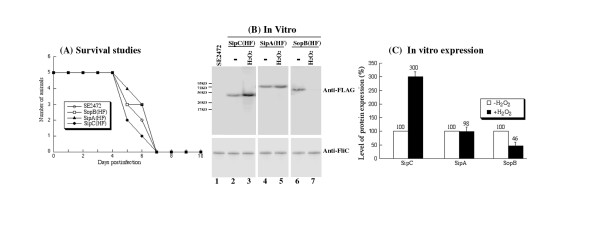
**(A) Mortality of BALB/c mice infected with *Salmonella *strains, (B) Western blot analyses of the synthesis of the tagged proteins from SE2472 (lane 1), SipC(HF) (lanes 2-3), SipA(HF) (lanes 4-5), and SopB(HF) (lanes 6-7), and (C) Effect of the treatment of hydrogen peroxide on the expression of the tagged SPI-1 proteins**. (A) Mice (5 animals per group) were infected intragastrically with 1 × 10^6 ^CFU of each bacterial strain. Mortality of mice was monitored for at least 10 days postinfection. (B) The expression of bacterial FliC was used as the internal control. The bacterial strains were grown in LB broth in the absence (-, lanes 2, 4, and 6) and presence of 5 mM H_2_O_2 _(H_2_O_2_, lanes 3, 5, and 7) at 37°C for 2 hours. SE2472 was grown in the absence of H_2_O_2 _(lane 1). Protein samples were separated in SDS-polyacrylamide gels and reacted with antibodies against the FLAG sequence (top panel) and FliC (low panel). Each lane was loaded with material from 5 × 10^7 ^CFU bacteria. The molecular masses of some of the proteins in the PageRuler protein size markers (Fermentas) are shown and given in kiloDaltons (KD). (C) Cultures of the tagged strains SipA(HF), SipC(HF), and SopB(HF) were grown in the absence and presence of 5 mM H_2_O_2_, as described in Methods and Materials. The values, which are the means from triplicate experiments, represent the relative percentage of the level of the tagged proteins from the bacteria grown in the presence of 5 mM H_2_O_2 _to those in the absence of H_2_O_2_.

To demonstrate the validity of our proteomic results, we examined the relative abundance of SipA, SipC, and SopB by Western blot analysis. *Salmonella *strains SipA(HF), SipC(HF) and SopB(HF) were derived from SE2472 and contained a FLAG epitope tag sequence at the carboxyl terminus of *sipA*, *sipC *and *sopB*, respectively [36]. The tagged strains grew in LB broth as well as the parental strain SE2472, indicating that the insertion of the tag sequence did not significantly affect bacterial growth *in vitro *[36](data not shown). To study the pathogenesis of the tagged strains in oral and systemic infection, we infected BALB/c mice intragastrically and intraperitoneally with the tagged *Salmonella *strains and compared infected mice to those infected with the wild type SE2472. The survival of infected mice and the colonization of spleen, liver, and ileum of the infected mice by *Salmonella *were determined at different time points post infection. For BALB/c mice infected intragastrically with 1 × 10^6 ^CFU of the tagged or the wild type strains, all infected mice died within 7 days post infection and no significant difference was observed among the wild type and the tagged strains (Figure 5A). No significant difference in the colonization of the internal organs such as spleen, liver, and ileum, was observed between the parental (wild type) SE2472 strain and the tagged strains regardless of the route of inoculation (Table 4). These results suggest that tagging of the target ORF does not impair the invasiveness, growth, and virulence of the bacteria, and that the tagged strains can be used as model strains to study infection of *Salmonella in vitro *and *in vivo*, including the expression of the SPI-1 proteins.

**Table 4:** The numbers of bacteria (CFU) in different organs from animals.

*Salmonella *strains	Colonization (i.p.)		Colonization (i.g.)	
	
	log CFU per organ		log CFU per organ	
	
	Liver	Spleen	Liver	Ileum
SE2472	9.0 ± 0.5	8.3 ± 0.5	9.1 ± 0.5	8.2 ± 0.5

SipA(HF)	9.1 ± 0.5	8.2 ± 0.5	8.9 ± 0.5	8.3 ± 0.5

SipC(HF)	9.2 ± 0.5	8.4 ± 0.5	9.0 ± 0.5	8.2 ± 0.5

SopB(HF)	9.0 ± 0.5	8.4 ± 0.5	9.2 ± 0.5	8.1 ± 0.5

* BALB/c mice were either infected intraperitoneally (i.p.) with 1 × 10^4 ^CFU or intragastrically (i.g.) with 1 × 10^6 ^CFU bacteria. A group of 5 mice was infected and the organs were harvested at 4 (for i.p. infection) or 6 days (for i.g. inoculation) post infection. Each sample was analyzed in triplicate and the analysis was repeated at least three times. The CFU of the sample was expressed as the average of the values obtained. The concentrations of bacteria were recorded as CFU/ml of organ homogenate. The limit of bacteria detection in the organ homogenates was 10 CFU/ml.

To determine the effect of H_2_O_2 _on the expression of the tagged ORFs, bacterial strains were grown in LB broth in the absence and presence of H_2_O_2_. Western blot analyses were used to determine the expression of the tagged proteins with an anti-FLAG antibody (Figure 5B, top panel). The expression of bacterial FliC protein, which was not significantly altered in the presence of 5 mM H_2_O_2 _(Table 2), was used as the internal control (Figure 5B, lower panel). Normalization of samples was also carried out by loading total proteins extracted from the same CFU (e.g. 5 × 10^7 ^CFU) of bacteria in each lane. Consistent with the results from our proteomic analyses (Table 2 and 3), the levels of SipC and SopB were about 3-fold higher and 2-fold lower in the presence of H_2_O_2_, respectively, while no change in the expression of SipA was detected (Figure 5B-C).

### Differential expression of SPI-1 factors in cultured macrophages and the spleen of infected animals

**Figure 6:**
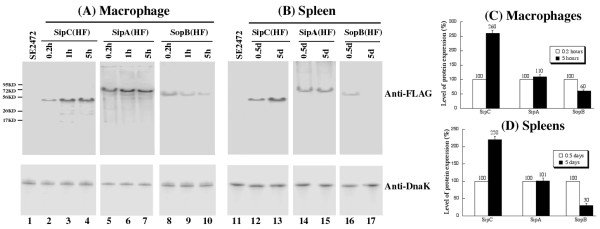
**Western blot analyses of the expression of the tagged proteins from bacterial strains SE2472 (lanes 1 and 11), SipC(HF) (lanes 2-4, 12-13), SipA(HF) (lanes 5-7, 14-15), and SopB(HF)(lanes 8-10, 16-17)**. In (A), bacterial protein samples were isolated from macrophages at 0.2, 1, and 5 hours of postinfection. In (B), BALB/c mice were intraperitoneally infected with 1 × 10^6 ^and 1 × 10^4 ^CFU of the tagged strains, and internalized bacteria were recovered from the spleen at 0.5 days and 4 days post inoculation, respectively. The expression of bacterial DnaK was used as the internal control. Protein samples were reacted with antibodies against the FLAG sequence (top panel) and DnaK (low panel). Each lane was loaded with material from 5 × 10^7 ^CFU bacteria. (C-D). Level of tagged proteins from the bacterial strains recovered from the macrophages and spleens of infected mice as determined in (A) and (B). The values, which are the means of triplicate experiments, represent the relative percentage of the levels of the tagged proteins in the bacteria recovered from macrophages (C) at 5 hours postinfection and from the spleen at 5 days postinoculation (D), as compared to those in the bacteria recovered from macrophages at 0.2 hours postinfection and from spleen at 0.5 days post inoculation, respectively.

Immunodetection of the SPI-1 proteins in cultured media in the absence and presence of H_2_O_2 _validated the proteomic observations. To evaluate the presence of these proteins in an environment more relevant to infection, the tagged *Salmonella *strains were used to infect macrophages and mice, and the expression of the tagged proteins was determined by immunodetection at different time points following infection. The expression of the tagged proteins in the bacterial strains isolated from the macrophages and the spleen of infected mice was detected using Western blot analysis with an anti-FLAG antibody and normalized using the expression of bacterial protein DnaK as the internal control (Figure 6A-B). Normalization of protein samples was also carried out by loading total proteins extracted from the same CFU (e.g. 5 × 10^7 ^CFU) of bacteria in each lane. The protein level of DnaK did not appear to be significantly different in bacteria recovered from macrophages [26], and from the spleen of infected animals as similar amount of the DnaK protein was detected from 5 × 10^7 ^CFU of each bacterial strain regardless of infection route (intraperitoneally or intragastrically) or time point postinfection (12-24 hours or 5-7 days)[16](data not shown).

In cultured macrophages, SipA, SipC, and SopB were all expressed at the early phase (e.g. 0.2 h) of infections. However, by 5 hr post infection, the levels of the three SPI-1 proteins diverged, with the SipC level increased, the SopB level decreased while SipA level remained unchanged (Figure 6A and 6C). To determine the relative abundance of these proteins in the spleen during systemic infection, BALB/c mice were infected intraperitoneally. *Salmonella *was recovered from the spleen at different time points postinfection, and the expression levels of the tagged proteins were determined. Similar to the results of macrophage infection, all three proteins were detected during the early stage of infection (i.e. 0.5 days). However, at a later stage of systemic infection (i.e. 5 days), the level of SipC increased and the level of SopB decreased while the level of SipA remained unchanged (Figure 6B and 6D). These results correlated with those observed in the proteomic analyses and in the macrophage experiments. Furthermore, these data strongly suggest that different SPI-1 factors are specifically expressed at late stage of *Salmonella *infection, and highlight a possible role of SipC in late phase of macrophage and *in vivo *infections of *Salmonella*.

## Discussion

### Stable isotope labeling procedure coupled with MS-based analysis for quantitative proteomic study of bacterial protein expression

In the postgenomic era, new methodologies are needed that can quantitatively, globally, and accurately measure protein expression in cells and tissues [37]. In this study, we have modified the SILAC method to develop a stable isotope labeling procedure coupled with MS analysis to carry out quantitative proteomic analysis of *Salmonella*. As a "proof of principle" pilot study, a total of 103 SE2472 proteins were monitored for their expression profiles upon exposure to H_2_O_2_.

At least seventy six proteins have been found to be modulated in the presence of H_2_O_2_. For example, the expression of SPI-1 proteins SipC and SopB was found to be differentially regulated in the presence of H_2_O_2_, while the expression of SipA remained unchanged. The level of SipC increased with H_2_O_2 _exposure, and the level of SopB decreased. These results were confirmed using Western blot analyses of protein expressions from FLAG-tagged *Salmonella *strains incubated with H_2_O_2_, validating the accuracy and reproducibility of our system for quantitative analyses of protein expression.

### Modulation of *Salmonella *protein expressions upon exposure to oxidative stress

Many *Salmonella *proteins we analyzed showed a moderate amount of up-regulation upon exposure to oxidative stress (Table 2 and 3), consistent with earlier studies involving *E. coli*'s response to oxidative stress [9-11,38]. For example, RecA (DNA strand exchange and recombinant protein) has been shown to be induced along with members of heat shock proteins [39]. The expression of superoxide dismutase SodB, which is a part of the SoxRS system [6,7,9], increased by 110%. When categorized by protein functions, we observed several patterns (Table 3). First, many enzymes involved in glycolysis and the TCA cycle were upregulated, showing up to a 330% increase. Consistent with the increase in general metabolism, amino acid biosynthesis was also affected in a positive fashion. Considering that intermediates from the glycolytic pathway are used in amino acid biosynthesis, the overall upregulation in downstream pathways is expected. This is consistent with our previous observations that amino acid supplementation increased the resistance of *E. coli *to H_2_O_2 _[38]. Interestingly, the pentose phosphate pathway was relatively unaffected in the presence of H_2_O_2_. Since one of the primary functions of the pathway is to generate ribose-5-phosphate for the synthesis of nucleotides and nucleic acids, other enzymes involved in nucleotide biosynthesis should show little change either. As expected, three such enzymes detected in this study (i.e. amidophosphoribosyltransferase, thymidine phosphorylase, and uridine phosphorylase) showed a varied response, ranging from a minor upregulation to a downregulation (Table 3). Further investigation of additional enzymes involved in the process should reveal the nature of this response.

We have noted that different proteins within the same operon may exhibit different expression levels in our results. Differential expression of proteins within the same operon has been reported [40] and may represent a regulatory mechanism for the expression of functional protein complexes. We have also noted that in some instances one protein was detected while another within the same operon was not. For example, redundant hydrogen peroxide scavenger systems have been reported to be present in *Salmonella *[41]. In our results, AhpC was not regulated while the other scavengers (KatE, KatG, KatN and TsaA) were not detected. One of the reasons for the divergence from expected protein level could be the limitation of the methodology we used in the study.

We used two-dimensional (2-D) gel electrohphoresis coupled with peptide fingerprinting by mass spectrometry which allowed us to perform global protein profiling quantitatively. However, our methodology is limited to proteins that can be detected by 2-D gel electrophoresis and identified by peptide fingerprinting. Proteins with low abundance or could not be identified by peptide fingerprinting for various reasons (e. g. post-translational modifications, resistance to trypsin digestion, or poor ionization of peptides) were not included in our analysis. Thus, our study by no means encompasses all the possible proteins expressed by SE2472 and we are presenting only the proteins we were able to successfully identify by peptide fingerprinting with high confidence in all three independent experiments. The absence of a protein in our results does not necessarily mean it was not expressed and/or induced; instead its expression status is yet to be determined. Our results are consistent with the notion that current proteomic approaches, including liquid chromatography mass spectrometry (LC-MS) and MALDI-ToF procedures, do not have the capacity to detect the entire proteomes of *Salmonella *[25-28]. Each approach has been shown to detect a distinct set of *Salmonella *proteins that exhibited limited overlap of protein coverage, and these complementary approaches should be carried out independently to generate a complete and full coverage of bacterial proteomes.

### Expression of SPI-1 proteins in post-invasion and late phase of *Salmonella *infection

Our proteomic results on SPI-1 proteins SipA, SipC, and SopB suggest that the expression of these proteins may be differentially modulated during infection under biologically relevant environments that resemble the oxidative stress condition. Efficient expression of SipA at late stage of infection in macrophages and in the spleen, as shown in our results, has been observed in *Salmonella enterica *serovar Typhimurium [15,16]. This is consistent with its functions in modulating actin dynamics and bacterial localization in infected macrophages [42-44] and in inducing inflammatory response for supporting *Salmonella *infection [45,46].

Our results of SopB protein expression are consistent with recent proteomic analysis results that *Salmonella enterica *serovar Typhimurium (strain 14028) reduced SopB protein expression by more than 2-fold within 4 hours of infection of RAW264.7-like macrophages [47]. SopB encodes a phosphoinositide phosphatase and is a multifunctional protein important for bacterial infection [48]. It facilitates bacterial invasion by inducing membrane ruffling and modulating actin polymerization [49-51], and stimulates inducible nitric oxide synthase (iNOS) production long after invasion and participates in the formation of the *Salmonella*-containing vacuole in macrophages [52-54]. Recently, SopB has been shown to carry out its diverse functions by localizing to different cellular compartments in a ubiquitin-dependent manner [48]. The reduced expression of SopB in the presence of H_2_O_2 _and at later time points of infection in macrophages and in the spleen in our study is consistent with the notion that SopB negatively regulates expression of sorting nexin 6 (SNX6), a protein involved in intracellular transport [47].

Our results also provide the first direct evidence that SipC is expressed in the spleen at late stages of *Salmonella enterica *serovar Enteritidis infection in mice. SipC is a *Salmonella *invasion protein (Sip) that is central for the initiation of the bacterial entry process. SipC and SipB form an extracellular complex following their secretion through the SPI-1 T3SS, and they are thought to assemble into a plasma membrane-integral structure (translocon) that mediates effector delivery [55-57]. Furthermore, SipC has been reported to promote actin nucleation and contribute to *Salmonella*-induced inflammation [58]. While the expression of SipC has been studied *in vitro*, its expression in the spleen has not been extensively investigated. The induced expression of SipC in *Salmonella *in the presence of oxidative stress and at late stages of infection in macrophages and in the spleen suggests that the level of this protein is highly regulated *in vivo *and that appropriate level of expression may contribute to the pathogenesis of *Salmonella*. This is consistent with recent observations that the translocase activity of SipC is important for the delivery of effector proteins and attachment of *Salmonella *to non-phagocytic cells; however, in the context of systemic infection, its actin-binding activity may facilitate bacterial infection of phagocytes [5,58,59]. Thus, examination of the expression of SipC and other SPI-1 factors both *in vitro *and *in vivo *in the context of infection, as reported in our study, is crucial to ultimately understand the actual functions and actions of these factors.

Using a different quantitative proteomic analysis approach without stable isotope labeling, Smith and co-workers have recently reported the protein expression of *Salmonella enterica *serovars Typhimurium and Typhi that grew in different culture conditions (e.g. stationary, log, and phagosome-mimicking conditions) and in macrophages [25-28]. Proteomic analysis of *Salmonella *protein expression in the spleen of infected animals has also been reported [24]. In these studies, the protein expression of the *S. Typhimurium *homologs of many of the oxidative stress-responsive proteins identified in our study were found to be modulated under phagosome-mimicking conditions and in macrophages, further validating our analysis as an accurate and reproducible approach for quantitative proteomic analysis. Some of our protein expression results may not be consistent with those of messenger RNA expression that have been recently published [19-23] as the expression of many *Salmonella *genes is tightly controlled both transcriptionally and post-transcriptionally [18,60]. Our results of protein expression *in vivo *may not necessarily correlate with the previous observations *in vitro *because of the different environments *Salmonella *was exposed to. The difference between our results and previously published reports may also be due to the difference in the serovars and strains used for the studies, and the coverage of the proteins due to different methodologies used for the studies [25-28,33]. None of these previous studies has reported the differential expression of SipA, SipC, and SopB in hydrogen peroxide-treated *Salmonella*, as described in our study. Our results complemented and further extended previous proteomic analysis of *Salmonella*, and furthermore, demonstrated the importance of examining the expression of *Salmonella *proteins, including SPI-1 proteins, *in vitro *using different quantitative proteomic analyses and *in vivo *in the context of infection.

Each of the currently-available proteomic approaches, including LC-MS and MALDI-ToF procedures, can only detect a subset of *Salmonella *proteins and may exhibit limited overlap of protein coverage with other methods [25-28]. It is suggested that these complementary approaches should be carried out independently to generate a comprehensive coverage of bacterial proteomes. Further investigation with our quantitative proteomic approach, in combination with examination and confirmation of the expression of these proteins *in vivo*, should provide significant insights into the role of these proteins in pathogenesis during *Salmonella *infection.

## Conclusion

We have employed stable isotope labeling coupled with mass spectrometry to carry out a quantitative proteomic analysis of *Salmonella enterica *serovar Enteritidis. Seventy-six proteins whose expression is differentially modulated upon exposure to H_2_O_2 _have been identified. SPI-1 effector SipC was expressed approximately 3-fold higher and SopB was expressed approximately 2-fold lower in the presence of H_2_O_2_, while no significant change in the expression of another SPI-1 protein SipA was observed. The expression of these SPI-1 factors was confirmed by Western blot analyses, validating the accuracy and reproducibility of our approach for quantitative analyses of protein expression. Furthermore, substantial expression of SipA and SipC but not SopB was found in the late phase of infection in macrophages and in the spleen of infected mice. This study provides the first direct evidence that SipC is highly expressed in the spleen at late stage of salmonellosis *in vivo*. Our results also suggest a possible role of the identified proteins, including SipC, in supporting the survival and replication of *Salmonella *under oxidative stress and during its systemic infection *in vivo*.

## Methods

### Reagents and preparation of protein samples for proteomic analysis

All reagents were obtained from Sigma-Aldrich unless otherwise specified. *Salmonella enterica *serovar Enteritidis (clinical isolate SE2472) [33] was cultured in LB broth-like normal (^14^N) and ^15^N-labeled media (Silantes GmbH, München, Germany), which are identical in chemical composition. The percentage of ^15^N in the labeled media is more than 98% (Silantes GmbH, München, Germany). The cultures were inoculated with a starter culture grown in normal (^14^N) or ^15^N-labeled media until mid-log phase. Two hundred fifty milliliter culture medium was inoculated with each starter culture and grown at 37°C with shaking at 225 rpm for 4 h. ^15^N-labeled culture was treated with 5 mM H_2_O_2_, which is well below the minimal inhibition concentration (MIC) of SE2472 (20 mM), and both cultures were grown for 2 h following the addition of H_2_O_2_. Protein extraction was performed with B-PER^® ^bacterial protein extraction reagent (Thermo Fisher Scientific, Rockford, IL) and quantified with D_c _Protein Assay Kit (Bio-Rad, Hercules, CA), which has an error rate of 2.5% in our experiments. We took this error rate into consideration by classifying any protein that had a 5% change or less as unchanged (having a 0% change).

### Two-dimensional gel electrophoresis and visualization of bacterial proteins

Protein samples were further solubilized in rehydration buffer (8 M urea, 2% CHAPS, 50 mM DTT, 0.2% Bio-Lyte^® ^3/10 ampholytes [Bio-Rad, Hercules, CA] and trace amount of Bromophenol Blue). ReadyStrip™ IPG strips (Bio-Rad, Hercules, CA) were loaded with 200 μg of protein samples (either normal or 1:1 mixture of normal and ^15^N-labeled samples) for preparative 2 D gels, and allowed to rehydrate for 18-22 h. Isoelectric focusing (IEF) was performed at 20°C using PROTEAN^® ^IEF cell (Bio-Rad, Hercules, CA). A 3-step protocol (250 V-20 min/8,000 V-2.5 h/8,000 V-10,000 V.h) was used for the IEF procedure following manufacturer's recommendations (Bio-Rad, Hercules, CA).

After the IEF procedure, the IPG strips were reduced in Equilibration Buffer I (6 M urea, 2% SDS, 0.375 M Tris-HCl [pH 8.8], 20% glycerol, 2% DTT) and alkylated in Equilibration Buffer II (6 M urea, 2% SDS, 0.375 M Tris-HCl [pH 8.8], 20% glycerol, 0.25% iodoacetamide). Strips were loaded onto 8-16% Criterion™ Tris-HCl SDS gel (Bio-Rad, Hercules, CA) and electrophoresed at 200 V for 65 min. Gels were visualized using Coomassie Brilliant Blue R-250 or silver staining (Invitrogen, Carlsbad, CA).

### Mass spectrometric identification of proteins

Gels were scanned and protein spots of interest were excised using the Xcise automated gel processor (Proteome Systems, North Ryde, Australia). Gel spots were destained and washed, followed by in-gel tryptic digestion using proteomic grade trypsin (Sigma-Aldrich, St. Louis, MO). Peptide fragments were collected and purified using ZipTip™ C_18 _reverse-phase prepacked resin (Millipore, Billerica, MA) and mixed with an equal volume of 10 mg/ml α-cyano-4-hydroxy-*trans*-cinnamic acid (Sigma-Aldrich, St. Louis, MO) in 0.1% trifluoroacetic acid (TFA)/50% acetonitrile solution and directly spotted onto a stainless steel target plate for mass analysis. Axima-CFR™ Plus (Shimadzu Biotech, Columbia, MD) was used for MALDI-ToF MS analysis, and 50-100 profiles were obtained for each sample, ensuring sufficient peak data for database interrogation. Probability-based scoring method with MASCOT database search engine (Matrix Science, Boston, MA) was used to identify each protein, based on the likelihood of search results being a random match. We used the following parameters for our protein identification: Database: NCBINR, MASCOT value cut off: greater than 62 (*p *< 0.05), Taxonomy: *Salmonella*, Missed cleavage: 1, Peptide Tolerance: +/- 0.75 Da, Variable modification: none, Fixed modification: none, Enzyme: Trypsin, Mass Values: Monoisotopic.

### Quantitative analysis

Tryptic peak data from MASCOT database searches was tabulated and elemental composition of each peptide fragment was determined using an in-house data analysis software. The process was further automated using a custom VBScript written for Microsoft Excel, which was designed to calculate predicted ^15^N peak location based on the primary amino acid sequence of tryptic peptide fragments. ^14^N/^15^N mixture MS spectrum was used to obtain peak intensity ratio between labeled (^15^N) and unlabeled (^14^N) samples to give relative quantification data. An average of 10 peaks was used to calculate the mean intensity ratios and the error percentage of each protein spot. Significant outliers were manually removed from the data set to prevent them from affecting the results (less than 2%). To further increase the accuracy of our results, experiments were preformed three times, and the results were the average from the triplicate experiments. Only those proteins that were detected and identified with high confidence in all three independent experiments are listed in Table 1 and Table 2.

### Growth and survival analysis of *Salmonella*

Strains SipA(HF), SipC(HF) and SopB(HF) are derivatives of the wild type *Salmonella enterica *serovar Enteritidis strain SE2472 with a FLAG tag inserted in-frame at the C-terminus of each corresponding protein and have been described previously [36]. Growth analysis of bacteria in LB or LB-like broth was carried out by first inoculating a single colony in 2 ml of either normal (^14^N) or ^15^N-labeled media and culturing at 37°C with shaking at 225 RPM overnight (about 16 hours) [16]. Thirty microliters of the overnight culture were then inoculated into 3 ml fresh normal or ^15^N-labeled media or LB broth and cultured at 37°C with shaking at 225 RPM. At 0, 2, 4, and 6 hours after inoculation, 100 μl of bacterial culture were collected to determine their colony forming unit (CFU)/ml by plating. *Salmonella *grew in normal (^14^N) or ^15^N-labeled media as well as in LB broth (data not shown). To study the survival of *Salmonella *after exposure to H_2_O_2_, 20 μl of the overnight culture grown in normal (^14^N) or ^15^N-labeled media, or LB broth were added to 2 ml of fresh normal (^14^N) or ^15^N-labeled media, or LB broth containing 5 mM H_2_O_2_. At different time points of incubation, 100 μl of bacterial culture were collected, diluted, and plated onto LB agar plates to determine their CFU/ml [16,36]. Each sample was analyzed in triplicates and the analysis was repeated at least three times.

### *In vitro *studies of the expression of the tagged SPI-1 proteins

Colonies of tagged strains were inoculated in 1 ml of LB broth and cultured at 37°C with shaking at 225 RPM for 16 hours. To study the effect of H_2_O_2 _on the protein expression *in vitro*, 20 μl of overnight bacterial cultures were inoculated into 1 ml of antibiotic-free LB and shaken at 225 RPM at 37°C for 4 hours. The bacterial cultures were centrifuged at 5,000 × g for 5 minutes. The pelleted bacteria were re-suspended in 1 ml of fresh LB broth (control) or 1 ml of LB broth with 5 mM H_2_O_2 _and shaken at 225 RPM at 37°C for an additional 2 hours, and then collected.

To prepare protein samples from *Salmonella*, bacterial cultures (1 ml) were centrifuged at 5,000 × g and 4°C for 10 minutes. The pellets were re-suspended in 200 μl of bacterial lysis buffer (8 M urea, 2% CHAPS, and 10 mM Tris, pH8.0), sonicated for 15 seconds three times with an interval of 30 seconds, centrifuged at 5,000 × g and 4°C for 10 minutes, and then transferred into fresh tubes for Western blot analysis.

### Infection of cultured macrophages

RAW264.7 macrophage-like cells (ATCC, Manassas, VA) were infected with stationary phase bacteria at a multiplicity of infection of 50. After incubation for 30 mins, infected cells were washed twice with phosphate-buffered saline (PBS) and incubated in DMEM medium supplemented with gentamicin (100 μg/ml) for 1 hour to eliminate extracellular bacteria. Then the cells were again washed twice with PBS, and incubated in DMEM supplemented with gentamicin (20 μg/ml). At various times postinfection, the cells were collected and resuspended in lysis buffer (120 mM NaCl, 4 mM MgCl_2_, 20 mM Tris-HCl [pH 7.5], 1%, Triton X-100) supplemented with protease inhibitors (complete EDTA-free cocktail, Roche Applied Science, Indianapolis, IN), incubated at 4°C for 1 hour, and centrifuged at 18,000 × g and 4°C for 10 minutes. The pellets that contained bacterial proteins were resuspended in PBS for Western blot analyses.

### *In vivo *studies

BALB/c mice (6-8 weeks old) were obtained from Jackson Laboratory (Bar Harbor, ME). Overnight bacterial cultures were serially diluted to suitable CFU/ml in PBS before infection. To assess the virulence of the tested strains, groups of five mice were either inoculated intragastrically with 1 × 10^6 ^CFU per mouse or intraperitoneally with 1 × 10^2 ^CFU per mouse. Mice were monitored during the course of infection, and those animals that exhibited extreme stress or became moribund were euthanized. For organ colonization experiments, groups of five mice were inoculated intraperitoneally with 1 × 10^4 ^or 1 × 10^6 ^CFU per BALB/c mouse of the bacterial strains, and were euthanized at 4 days or 12 hours after inoculation, respectively. Mice were also intragastrically infected with 1 × 10^6 ^CFU per BALB/c mouse of the bacterial strains, and were euthanized at 6 days after inoculation. Organs were collected and homogenized in PBS at 4°C. An aliquot of each homogenate was used to determine its CFU/ml by serial dilution with PBS and plating onto LB agar plates. Each sample was analyzed in triplicate and the analysis was repeated at least three times. The CFU of the sample was expressed as the average of the values obtained. The concentrations of bacteria were recorded as CFU/ml of organ homogenate. The limit of bacteria detection in the organ homogenates was 10 CFU/ml. To prepare protein extracts for Western blot analyses, the homogenates of the spleen samples were centrifuged and the pellets that contained the bacteria were resuspended in PBS, following the procedures described previously [16]. All the experimental procedures with animals were in compliance with the guidelines and policies of the Animal Care and Use Committee (ACUC) of the University of California at Berkeley, and have been approved by the ACUC.

### Western blot analyses

The denatured polypeptides from bacterial lysates were separated on SDS-containing 10-12% polyacrylamide gels cross-linked with *N*, *N*''-methylenebisacrylamide (0.05%), transferred electrically to nitrocellulose membranes (Bio-Rad, Hercules, CA), and reacted in an enzyme-linked immunoassay with a monoclonal anti-FLAG antibody (Sigma, St Louis, MO) and antibodies against *Salmonell*a FliC (BioLegend, San Diego, CA) and DnaK (StressGen, Victoria, British Columbia, Canada), followed by an anti-mouse IgG conjugated with alkaline phosphatase [16,36]. The membranes were subsequently stained with a chemiluminescent substrate with the aid of a Western chemiluminescent substrate kit (Amersham Inc, GE Healthcare) and quantified with a STORM840 phosphorimager. Normalization of samples was also carried out by loading total proteins extracted from the same CFU (e.g. 5 × 10^7 ^CFU) of bacteria in each lane.

## Authors' contributions

KK, EY, GV, HG, JS, FL, and SL conceived the study, performed the research, analyzed the results, and wrote the paper. All authors read and approved the final manuscript.
